# Restructuring Patient Review and Allocation in a South London Home Treatment Team

**DOI:** 10.1192/bjo.2022.282

**Published:** 2022-06-20

**Authors:** Theo Boardman-Pretty, John Tweed, Camilla Day, Lucy Stephenson, Jalon Quinn, Larry Rifkin

**Affiliations:** South London & Maudsley NHS Trust, London, United Kingdom

## Abstract

**Aims:**

Lambeth Home Treatment Team (LHTT) provides short-term intensive community psychiatric care to a diverse South London population. The high turnover of patients requires a streamlined process to review and discuss their progress. We aimed to discuss patients in more frequent, targeted and shorter meetings, and to improve continuity of medical care using a ‘named doctor’ system. We assessed impact on length of stay with LHTT, on staff time as well as on both patient and staff satisfaction.

**Methods:**

The system of once-weekly day-long discussions of entire caseload was replaced by twice-weekly discussions of new and concerning patients only. The system of medical reviews was changed from ad hoc to MDT-agreed allocation to a specific doctor for the duration of LHTT stay.

Data on duration of treatment and caseload size were taken from regular LHTT statistical reports. Staff and patient questionnaires assessed impact on satisfaction and time spent in review discussions.

**Results:**

Qualitative reports of staff experience revealed that the new system was felt to provide better continuity of care, better time efficiency (less time spent learning about new patients) and improved learning experiences for doctors in training. Downsides included lack of ‘automatic second opinion’ when a patient was reviewed by a different doctor, felt to be mitigated by more frequent discussions in MDT reviews when needed.

Patient feedback showed no significant change was noted in overall experience of LHTT, although patients were more likely to feel involved in their care (88% said ‘definitely’ compared to 68% before the change).

Time spent discussing patients in clinical review meetings reduced from an average of 38.5 to 28.5 person-hours per week.

Average caseload reduced from 57 to 42. However, duration of treatment increased from 18.8 days to 20.4 days.

**Conclusion:**

The reduction in staff time in reviews suggests that the system had been appropriately streamlined. While caseload size reduced, duration of stay slightly increased, so the new system was not found to have had a significant impact on objective measures of patient care.

Staff feedback was generally favourable, highlighting continuity of care and time efficiency. Patient feedback, while good both before and after our change, suggested a greater feeling of involvement in their care, possibly due to clearer communication and discussion of plan from the start of LHTT care.

